# *Centella asiatica* ameliorates AlCl3 and D-galactose induced nephrotoxicity in rats via modulation of oxidative stress

**DOI:** 10.6026/973206300200508

**Published:** 2024-05-31

**Authors:** Thirupathirao Vishnumukkala, Prarthana Kalerammana Gopalakrishna, Barani Karikalan, Warren Thomas, Saravanan Jagadeesan, Samaila Musa Chiroma, Nurul Huda Mohd Nor, Mohamad Aris Mohd Moklas

**Affiliations:** 1 Department of Human Anatomy, Faculty of Medicine and Health Sciences, Universiti Putra Malaysia, Serdang, Selangor, Malaysia; 2 Anatomy discipline, Human Biology Division, School of Medicine, IMU University, Kuala Lumpur, Malaysia; 3 Physiology discipline, Human Biology division, School of Medicine, IMU University, Kuala Lumpur, Malaysia; 4 Department of Pathology, Faculty of Medicine, Bioscience and Nursing, MAHSA University, Bandar Saujana Putra, Selangor, Malaysia; 5 Department of Human Biology, Royal College of Surgeons in Ireland - Medical University of Bahrain, Al Sayh, Muharraq Governate, Kingdom of Bahrain; 6 Department of Anatomy, School of Medicine, Taylors University, Lakeside Campus, Selangor, Malaysia; 7 Newcastle University Medicine Malaysia, Iskandar Puteri, Johor, Malaysia

**Keywords:** *Centella asiatica*, Nephrotoxicity, AlCl3, D-galactose, Oxidative stress

## Abstract

Nephrotoxicity is a condition caused by toxic effects of medications and poisons resulting in the rapid decline of kidney function. *Centella asiatica* is a medicinal herb with antioxidative and anti-inflammatory characteristics that is used to treat a variety of ailments. The present study intends to explore the ability of *Centella asiatica* in preventing AlCl3 and D-Galactose-induced nephrotoxicity in rats. In this study 30 male albino Wistar rats were induced with nephrotoxicity using AlCl3 and D-galactose, and oral administration of *Centella asiatica* extract (100, 200, and 300mg/kg/day) was administered for 70 days. The kidneys were extracted after treatment and levels of oxidative and antioxidative enzymes, serum creatinine, and serum albumin were measured. The kidney's histopathological changes were studied. Administration of *Centella asiatica* extract significantly increased serum albumin, superoxide dismutase (SOD), and catalase levels in kidney homogenates while suppressing serum creatinine and malondialdehyde (MDA) levels and attenuating histopathological changes associated with nephrotoxicity. *Centella asiatica* extract lowered serum creatinine and oxidative stress levels in a drug-induced nephrotoxicity rat model, while simultaneously increasing serum albumin levels, as evidenced by mitigation of histological changes and normalisation of biomarkers of oxidative stress in the kidney.

## Background:

Kidneys are paired organs that have key functions in human health, playing a vital role in control of blood pressure, the pH of the extracellular fluid, and maintaining the balance of solutes and electrolytes in the plasma. [1] The kidneys perform a critical function in the elimination of xenobiotics from the body, which may be nephrotoxic compounds that can accumulate in the excreting organ in relatively large concentrations. [2] The term " drug-induced nephrotoxicity" refers to kidney damage caused by drugs or chemicals, resulting in pathophysiological effects such as lower glomerular filtration rate (GFR), hydro-electrolytic diseases (HED), and nephrotic syndrome. These effects can harm the acute and long-term health of the kidneys. [3] Nephrotoxicity is the third most common aetiology of Acute Kidney Disease (AKD), the incidence of which has increased in recent decades; the increase has been attributed to the increased use of nephrotoxic medications and lifestyle choices that may damage the kidneys. [4]. Several pathways have been implicated in causing nephrotoxicity, these include altered glomerular haemodynamics, toxicity affecting tubular cells followed by inflammation, crystal nephropathy, rhabdomyolysis and also thrombotic microangiopathy [5]. Aluminium (Al) is a reactive pollutant and exposure to it in industrial environments is a well-known cause of nephrotoxicity. Al is frequently present in various food products and widely distributed in different environments. [6] Epidemiological studies have conclusively shown that metal toxicants like Al contribute to chronic kidney damage, as the kidneys eliminate Al compounds from the body that were taken in through polluted sources such as food, water, or inhalation, so the high renal concentrations lead to pronounced nephrotoxicity. [7] The in vitro and in vivo studies conducted to investigate the effects of Al have revealed that Al's negative impact on cellular function arises by way generating reactive oxygen species (ROS) such as superoxide anions, hydrogen peroxide, and hydroxyl radicals. These ROS impair normal functioning of mitochondria, leading to an overabundance of highly reactive free radicals and electrons [8]. The serum levels of biomarkers for nephrotoxicity such as alanine aminotransferase (ALT), alkaline phosphatase (ALP), urea, and creatinine are all raised by Al exposure. [9] Reports from recent studies have suggested that neutralizing ROS is a crucial strategy to prevent pathological processes linked to Al exposure, and antioxidants treatment can reduce ROS levels and consequently prevent oxidative stress-related damage. [10] D-galactose (D-gal) is an abundant carbohydrate monomer metabolized as a reducing aldose after conversion to glucose. [11] Excessive D-gal intake can exceed the body's metabolic capacity, producing advanced glycation end products (AGEs) that can accumulate and attach to AGE receptors, causing damage to cells through free radical production, oxidative stress, apoptosis, and inflammation. [12] *Centella asiatica* (CA), a member of the Umbelliferae (Apiaceae) family, has been widely used as a medicinal plant in China, India, and Sri Lanka for thousands of years. CA contains a pentacyclic triterpenoid- Asiatic acid (AA), flavonoids, madecassoside, amino acids, brahmoside, glycosides, isothankuniside, and metastatic fatty acids that are the main active ingredients in CA extracts. [13] Studies have confirmed the nephroprotective effect of CA extracts on various renal diseases including cisplatin-induced acute renal failure, and in renovascular hypertensive rats. [14] Results from another study investigating urethral ligation induced nephrotoxicity have shown that treatment with CA extract effectively ameliorates kidney damage in rats. [15] However, a gap exists regarding the potential therapeutic benefits of CA in treating nephrotoxicity induced by AlCl3 and D-gal. Therefore, it is of interest to investigate the nephroprotective potential of CA in kidney injury induced by AlCl3 and D-gal in adult male Wistar rats, with a focus on its effects on the levels of serum albumin and creatinine, oxidative stress biomarkers, and histological alterations commonly observed in nephrotoxicity.

## Materials and Methods:

## Animals:

Thirty, three months old albino male Wistar rats (weighing 220-250g) were procured from a local supplier in Malaysia. The rats were housed and cared for in cages at the animal house at Universiti Putra Malaysia (UPM) with two animals per cage in a temperature-controlled facility with 12-hour alternate day/ night cycles. The animals were given regular food pellets (Harlan, UK) and access to water ad libitum. Ethical approval was obtained from the Institutional Animal Care and Use Committee (IACUC) at UPM bearing the certificate number UPM/IACUC/AUP-R071/2020. Every experiment was carried out as per the rules of UPM and IACUC.

## Plant extract and Chemicals:

The extract of CA was purchased from Universiti Teknologi MARA (UiTM), Malaysia, and D-galactose and AlCl3 were procured from Sigma Aldrich, USA. Chemicals of analytical grades were used in the experiments. Distilled water was used to dissolve D-galactose for intraperitoneal (i.p.) injection, as well as AlCl3 and CA extract for oral administration.

## Experimental design:

**Figure 1:**
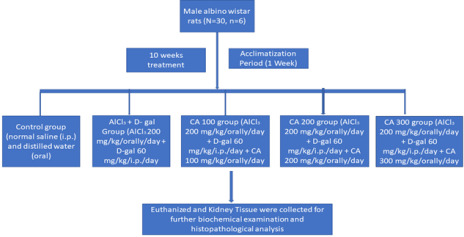
The study consisted of five groups: control, model (AlCl3 + D-galactose), CA 100, CA 200, and CA 300. Each group consists of six rats. The rats were subjected to decapitation following a 70-day period of treatment.

After one week of acclimation, the rats were randomly divided into five groups (n = 6) and treatments administered for a period of 10 weeks (Figure 1).

## Sample Collection and Preparation:

At the end of experimental period, blood samples were collected from the tail veins before euthanasia of the rats. After euthanisation the kidneys were removed and washed in ice-cold saline. The kidney samples meant for biochemical analysis were washed thoroughly in ice-cold phosphate buffer saline (PBS) and then stored at -80 °. The kidney samples taken to study the histological changes were cleaned with normal saline before being fixed for a week in 10% formalin solution, after which they were processed, and the histological changes were observed.

## Histopathology of kidney tissues:

Rat kidneys preserved in 10% formalin for one week, were washed, processed, encased in paraffin wax, and sectioned to a thickness of 5 µm using a microtome (Rotary Microtome RF-600). The sections were stained with haematoxylin and eosin. The histopathological changes were examined using an Olympus BX43 manual system microscope. Images were captured using an Image Analyzer (Nikon H500L) at a 40X magnification.

## Measurement of lipid peroxidation and antioxidant enzymes:

To evaluate the biomarkers of lipid peroxidation (LPO) in rat kidney homogenates, levels of the following biomarkers: malondialdehyde (MDA), superoxide dismutase (SOD), catalase (CAT), and glutathione peroxidase (GPx) were measured. MDA is a byproduct of LPO and Ohkawa *et al.* [16] described the method of using thiobarbituric acid (TBA) reagent for measuring MDA levels. The measurements were expressed as moles of MDA produced per mg of protein. The antioxidant enzyme activity in rat kidney homogenates was further evaluated by measuring SOD levels using the method described by Fridovich and Misra. [17] The SOD abundance was reported in units per mg of protein per minute.

## Measurement of serum creatinine and serum albumin:

Blood samples collected from the experimental rats were centrifuged at 3000 rpm for 5 minutes separating serum and other blood components. The separated serum was frozen to maintain its integrity. It is essential to use the serum for kidney function analyses within 12 hours of collection to ensure accurate results. The levels of serum creatinine were measured using colorimetric assay kit (Cayman Chemical Company, No. 700460) according to the manufacturer's instruction. The rat albumin ELISA Kit (Abcam, ab108789) was used to evaluate albuminuria. This test quantifies the albumin in the serum, an important marker of kidney function.

## Statistical Analysis:

The values presented in the results were expressed as mean ± standard deviation (SD) (n=6). The data was analyzed using one-way ANOVA and Tukey's post hoc test. While comparing treatments, the p-value differences of less than 0.05 were statistically significant. GraphPad Prism (version 9) software was used for the data testing.

## Results:

## Effect of CA extract on AlCl3 and D-gal induced nephrotoxicity by measurement of serum creatinine and albumin levels:

**Figure 2:**
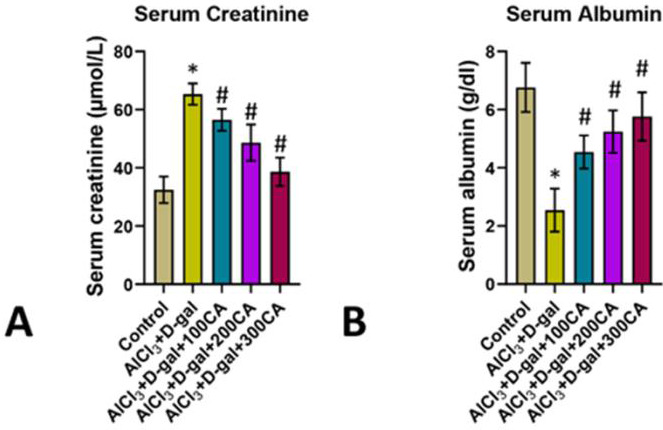
Represents the serum creatinine and serum albumin in the kidneys of experimental rats. (A) Serum creatinine; (B) serum Albumin. Data are expressed as mean ± S.D, n = 6, *p < 0.05 versus control and AlCl3 + D-gal; #p < 0.05 versus AlCl3 + D-gal + CA treatment.

Rats administered with AlCl3 and D-gal exhibited a notable increase in serum creatinine and decrease in albumin levels which was statistically significant compared to the untreated control group. This indicated potential kidney dysfunction or damage. However, when rats were treated with extracts of CA at dosages of 100mg, 200mg, and 300 mg/kg body weight at the same time as the nephrotoxic treatment, there was a discernible decrease in the elevated serum creatinine levels caused by AlCl3 and D-gal, which were now similar to the untreated group of rats. Similarly, the reduced serum albumin levels of the AlCl3 and D-gal treated rats were recovered back to the normal levels of the control when CA extract was co-administered (Figure 2).

## Effect of CA on nephrotoxicity induced by AlCl3 and D-gal on LPO pathways, MDA, SOD, catalase and GPx:

**Figure 3:**
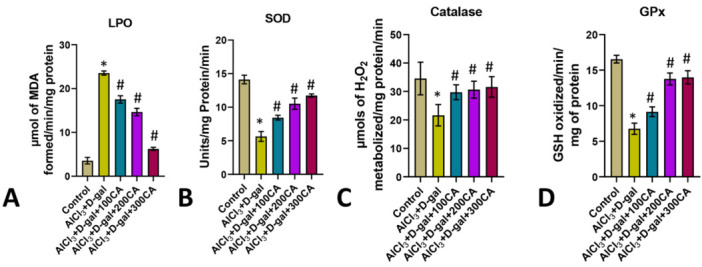
The oxidative and antioxidant enzymes in the kidneys of experimental rats are represented. (A) Lipid Peroxidation; (B) Superoxidase Dismutase; (C) Catalase; and (D) Glutathione Peroxidase. *p 0.05 versus control and AlCl3 + D-gal; #p 0.05 versus AlCl3 + D-gal + CA treatment; data are expressed as mean S.D, n = 6.

The kidneys of rats treated with AlCl3, and D-gal had substantially more abundant in MDA due to increased lipid peroxidation compared to the control group. MDA levels in AlCl3 and D-gal exposed rats which were co-administered with CA at dosages of 100mg, 200mg, and 300 mg/kg body weight, were substantially lower than those rats exposed to AlCl3 and D-gal (Figure 3A). Furthermore, the kidneys of rats exposed to AlCl3 and D-gal treatment had significantly lower levels of antioxidant enzymes like SOD, while rats that were co-administered with CA at dosages of 100, 200, and 300 mg/kg body weight showed a significant increase in levels of SOD (Figure 3B). Additionally, the kidneys of rats treated with AlCl3, and D-gal showed a substantial reduction in the levels of antioxidant enzyme catalase, which was attenuated in rats that were co-administered with extract of CA at 100, 200, and 300 mg/kg body weight (Figure 3C). A similar observation was made in the levels of catalase (GPx) another antioxidant enzyme in the kidneys of rats given AlCl3 and D-gal, which showed reduced levels of GPx, which was again recovered in rats co-administered with extracts of CA at 100, 200, and 300 mg/kg body weight (Figure 3D).

## Effect of CA on AlCl3 and D-gal induced nephrotoxicity as observed in histopathology:

The control rats exhibited the normal histology of the kidney with clear visualization of Bowman's capsule, proximal and distal convoluted tubules (Figure 3). The AlCl3 and D-gal treated group showed glomerular atrophy, degeneration, and tubular necrosis. Histological sections of kidney from rats co-administered with CA revealed alterations in basement membrane thickening, normal mesenchymal density, and decreased glomerular capillary degeneration due to enlarged Bowman's gap.

## Discussion:

The present study was conducted to establish the effect of CA extracts in attenuating AlCl3 and D-gal induced renal damage. Our data showed substantial changes in renal function and presence of histopathological aberrations after exposure to AlCl3 and D-gal that were consistent with increased renal oxidative stress. Co-administration of an extract of CA with AlCl3 and D-gal showed substantial improvement in all parameters related to renal function and structural alterations seen in histology. These data indicate that CA can ameliorate nephrotoxicity induced by AlCl3 and D-gal, by raising the levels of endogenous protective antioxidants. Various mechanisms have been suggested to regulate the renal excretion of Al from the body which includes glomerular filtration, reabsorption of filtered Al at the proximal tubules and active secretion in the distal part of nephrons. [18] Depending on the route of exposure, the kidneys may be subjected to high concentrations of Al during the normal process of renal excretion, thus rendering the kidney susceptible to Al mediated toxicity. [19] Most published research has examined the harmful effects of AlCl3 in animals following parenteral treatment, which does not necessarily mimic the primary pathway of exposure in humans or the levels of Al3+ experienced during renal excretion. [20] Previous studies have indicated that D-gal initiates oxidative damage in the liver and kidneys of rats. [21] Free radicals damage vital components of cells, through lipid peroxidation, which harms the cell membranes and organelles of cells in the liver and kidney. These membrane actions cause swelling and necrotization of hepatocytes and tubular cells, which culminates in a decline in liver and kidney function. [22] D-gal weakens immunological responses, lowers antioxidant enzyme activity, and promotes the generation of free radicals. [23] Increasing evidence suggests that oxidation and inflammation are closely related since free radicals are agents of inflammation released by macrophages and neutrophils while at the same time ROS released by tissues can initiate inflammatory responses. ROS production by which ever mechanism can cause an inflammatory damage to tissues when it is out of control. [24] Renal toxicity was induced in the rat model by administering AlCl3 orally and D-gal intraperitoneally for a period of 70 days, and simultaneously test groups were co-administered with different dosages of CA orally. The results of present study revealed that AlCl3 and D-gal induced increases in levels of serum creatinine. These findings are in accord with previous studies which reported that Al can contribute to renal injury, leading to many clinical disorders. [25] Renal tubules are the main sites of renal injury in rats exposed to AlCl3 over an extended period. [26] As the kidneys excrete AlCl3, there is a noticeable deterioration in tubular structure and function. A critical buildup of Al in the kidneys can lead to elevated serum urea and creatinine, indicative of renal failure. [27] Since the kidneys are the primary organs for elimination of numerous toxins, pollutants, and xenobiotics from the body, they are more likely to be exposed to large amounts of free radicals, which raise the oxidative stress levels in the kidney and play a pivotal role in the pathophysiology of various kidney disorders. [28] Consequently free-radical production can be implicated in the nephrotoxicity of AlCl3. [29] Al may decrease the activities of various tissue antioxidant defence system components, including GSH and SOD, which can increase the generation of free radicals, particularly ROS, and cause lipid peroxidation. [30] The kidney is one of the critical organs metabolizing D-gal. During D-gal therapy for congenital glycosylation (CDG) disorders, MDA levels rise and creates oxidative stress in the kidney. [31] In the widely used animal models for kidney damage, mice or rats are treated with D-gal for a period of 70 days. [32] This chronic exposure to D-gal in animal models elevated hepatic and renal MDA levels, oxidative stress leading to hepatopathy and nephropathy. D-gal exposure also markedly reduced renal SOD and CAT activity, and GSH abundance. [33] The protective effects of CA and its impact on D-gal-induced kidney injury is not well understood. In this study, co-administration of extract of CA to AlCl3 and D-gal treated rat's effectively enhanced renal function, as deduced from 1) reduced levels of blood creatinine and increased levels of serum albumin 2) reduced oxidative stress parameters 3) decreased histopathological changes of AlCl3 and D-gal induced nephrotoxicity. While serum indicators like creatinine may not be suitable for early kidney injury diagnosis, they help estimate overall damage to renal cells. [34] High serum creatinine and low serum albumin levels were observed in mice treated with AlCl3 and D-gal and abnormalities of these renal function indicators are typically the consequence of damage to the nephron structure, which impairs the kidney's ability to filter waste products and recover nutrients. [35] This study revealed abnormal levels of kidney function biomarkers in rats administered AlCl3 and D-gal. However, consistent with previous studies co-administration of CA extract prevented these abnormalities. [35] [36] Data shows that CA significantly reduced kidney histological structural disruption, degeneration, necrosis, and inflammatory cell infiltration in rats. Our findings demonstrated that increasing the dosage of CA further reduced the severity of renal injury in AlCl3 and D-gal induced rats. CA also reduced lipid peroxidation and boosted antioxidant enzyme activity in AlCl3 and D-gal-induced animals. It is generally recognized that the antioxidant enzyme defence system, which includes SOD, CAT, and GPx, can reduce oxidative stress and perhaps help oxidative stress-related disorders. [37] CA restored the antioxidant defence system operating in the kidneys of AlCl3 and D-gal exposed animals by boosting the levels and performance of antioxidant enzymes (SOD, CAT, and GPx) and reducing the levels and effects of lipid peroxidation (MDA). CA may help to control the pro-oxidant-antioxidant imbalance, and so prevent kidney injury.

## Conclusion:

Data shows that AlCl3 and D-gal-induced oxidative stress significantly creates an imbalance between the oxidant and antioxidant systems, serum creatinine, and serum albumin, resulting in cellular damage in the renal tubules. This effect is alleviated by co-administration of an extract of CA. CA extracts have an anti-oxidative effect due to free radical scavenging activity. Further research is needed to fully establish the mechanisms behind CA's control of oxidative stress markers in rats exposed to nephrotoxic agents. CA has the potential to be an effective agent for the treatment of drug-induced nephrotoxicity in the future.

## Figures and Tables

**Figure 4:**
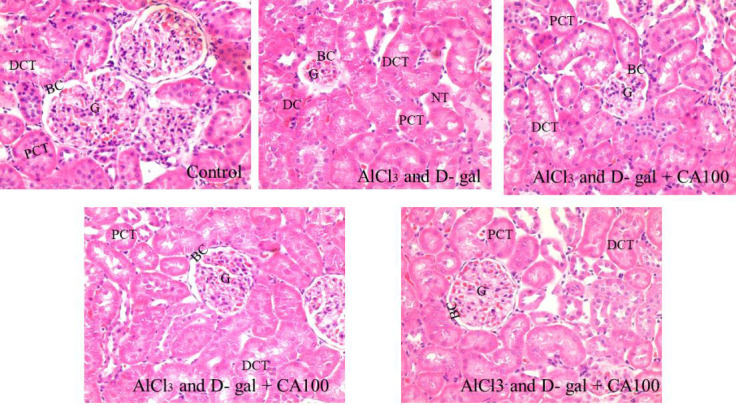
The control kidney has usual architecture, with substantial Bowman's capsule, epithelial cells, and normal tubules. Nephrotoxicity is shown by glomerular atrophy, degeneration, and tubular necrosis in the model. CA-treated sections have no atrophy, less degeneration than the model, normal tubules, and a distinct Bowman's capsule. (G: Glomerulus; BC: Bowmen's capsule; PCT: Proximal convoluted tubules; DCT: Distal convoluted tubules; NT: Necrotic tubules; DC: Dead cells)
